# Molecular and Morphological Investigations of Two Giant Diatom *Cymbella* Species from the Transbaikal Area (Russia, Siberia) with Comments on Their Distributions

**DOI:** 10.3390/plants11182445

**Published:** 2022-09-19

**Authors:** Anton M. Glushchenko, Yevhen I. Maltsev, John Patrick Kociolek, Irina V. Kuznetsova, Maxim S. Kulikovskiy

**Affiliations:** 1 K.A. Timiryazev Institute of Plant Physiology RAS, IPP RAS, 35 Botanicheskaya St., 127276 Moscow, Russia; 2 Museum of Natural History, Henderson Building, 15th and Broadway, Boulder, CO 80309, USA

**Keywords:** Bacillariophyceae, diatoms, new species, *Cymbella*, Russia, Transbaikal area, morphology, molecular investigation

## Abstract

For the first time, a giant diatom species of the genus *Cymbella* from Lake Baikal was studied using molecular methods. Molecular and morphological investigations allowed to us to described one new species, *Cymbella baicalaspera* Glushchenko, Kulikovskiy and Kociolek sp. nov. This species is both morphologically similar and phylogenetically close to a second giant *Cymbella* species that we investigated here, identified by us as *Cymbella himalaspera* Jüttner and Van de Vijver in Jüttner et al. 2010. This species was first described from Nepal on the basis of a morphological investigation. Small morphological differences exist between the type population and specimens from Lake Baikal, but otherwise the two are identical. These very interesting results show that some Baikalian diatoms can be distributed more widely and are not only endemic to this ancient lake. Similarity between *Cymbella baicalaspera* sp. nov. and *Cymbella himalaspera* on the basis of both morphological features and their close phylogenetic relationships suggested by molecular data indicate they are sister species and an example of sympatric speciation. These results also suggest an early development of a species flock. This species group warrants additional research in terms of. their diversification and biogeography.

## 1. Introduction

The genus *Cymbella* C.A. Agardh was proposed in 1830 [1]. The genus, which previously included a large number of taxa with dorsiventral symmetry, is currently divided into several genera, such as *Afrocymbella* Krammer, *Cymbopleura* (Krammer) Krammer, *Delicata* Krammer, *Encyonema* Kützing, *Encyonopsis* Krammer, *Gomphocymbellopsis* Krammer, *Navicymbula* Krammer, *Oricymba* Jüttner, Krammer, Cox, Van de Vijver and Tuji, *Pseudencyonema* Krammer, *Karthickia* Kociolek, Glushchenko and Kulikovskiy and *Vladinikolaevia* Kulikovskiy, Glushchenko, Y. Liu and Kociolek and *Qinia* Y. Liu, Kociolek and Kulikovskiy [2,3,4,5,6,7,8].

Taxa from the genus *Cymbella* are quite variable in valve length: from five microns to several hundred microns [2,5]. Fairly large-celled species of the genus include *C. amelieana* Van de Vijver and Lange-Bertalot, *C. aspera* (Ehrenberg) Cleve, *C. peraspera* Krammer, *C. himalaspera* Jüttner and Van de Vijver, *C. subhimalaspera* Jüttner and Van de Vijver, *C. halophila* Krammer, *C. amoyensis* Voigt, *C. cantonensis* Voigt, *C. bourrellyi* Maillard, *C. lanceolata* var. *bottnica* Krammer, *C. neolanceolata* Silva [2,9,10,11,12].

Recently, large-cell species *Cymbella* from Lake Baikal were described, including *C. amplificata* Krammer, *C. paraintermedia* Kulikovskiy, Metzeltin and Lange-Bertalot, *C. baicalensis* f. *cuneata* Kulikovskiy and Lange-Bertalot and *C. baicaloaustralica* Kulikovskiy, Metzeltin & Lange-Bertalot [13]. The Transbaikal area and its waterbodies are poorly studied. For example, very little molecular data exist on the diatoms from Lake Baikal and surrounding waterbodies; however, this information is important for understanding several important biological and ecological processes, including species diversity, presence of cryptic taxa, application of new tools for water quality assessment, and voucher cultures and sequence data about the organisms from this long-lived system and world-renowned lake [14,15,16,17,18,19,20,21,22,23,24].

The aim of this publication is the molecular investigation and description of the morphology of three strains of giant *Cymbella* taxa from Lake Baikal with description of one new species and a biogeographical note on *C. himalaspera* Jüttner and Van de Vijver.

## 2. Results

### 2.1. Molecular Investigation

**Figure 1:**
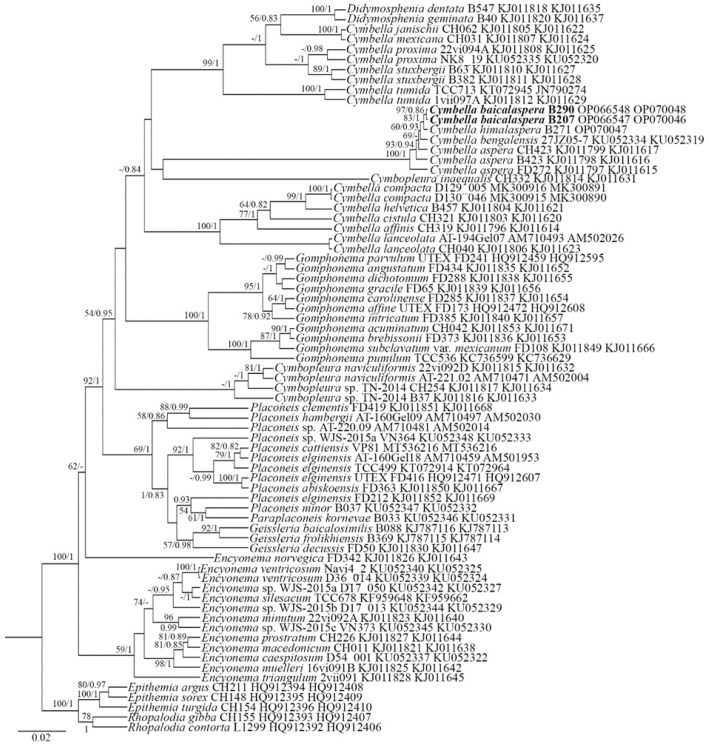
Phylogenetic position of *Cymbella baicalaspera* (indicated in bold) based on Bayesian inference for the partial 18S rDNA and *rbc*L genes. The total length of the alignment is 1027 characters. Bootstrap supports from ML (constructed by RAxML) and posterior probabilities from BI (constructed by Beast) are presented on the nodes. Only likelihood bootstraps and posterior probabilities above 50 and 0.85 are shown. Strain numbers (if available) and GenBank numbers are indicated for all sequences.

The three investigated strains, namely, *Cymbella baicalaspera* sp. nov. (B290 and B207) and *C. himalaspera* (B271), are shown to form a monophyletic group, and the evidence of them being sister species has high statistical support (ML 83; BI 1). Species closely related to our newly investigated strains are *C. bengalensis* and three strains identified as *C. aspera* (Figure 1). All these species form a monophyletic group and are closely related to other strains of *Cymbella* and *Didymosphenia*.

### 2.2. Morphological Investigation

**Figure 2:**
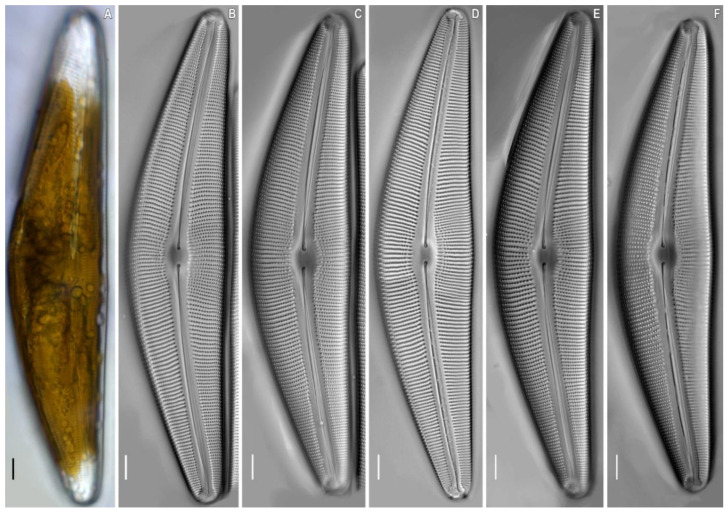
*Cymbella himalaspera* Jüttner and Van de Vijver in Jüttner et al. 2010. Strain B271. Light microscopy, differential interference contrast, size diminution series. (**A**) Live cells with chloroplast structure. (**B**–**F**) Oxidized material. Slide No. B271. Scale bars = 10 μm.

**Figure 3:**
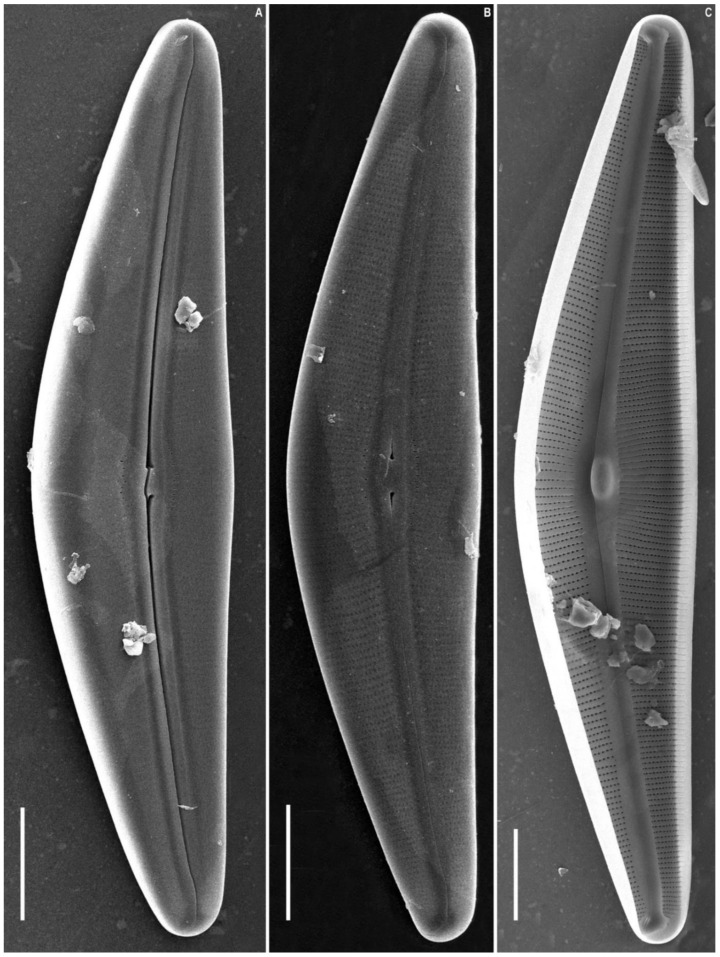
*Cymbella himalaspera* Jüttner and Van de Vijver in Jüttner et al. 2010. Strain B271. Scanning electron microscopy. (**A**,**B**) External views. (**C**) Internal view. Scale bars (**A**,**B**) = 25 μm; (**C**) = 20 μm.

**Figure 4:**
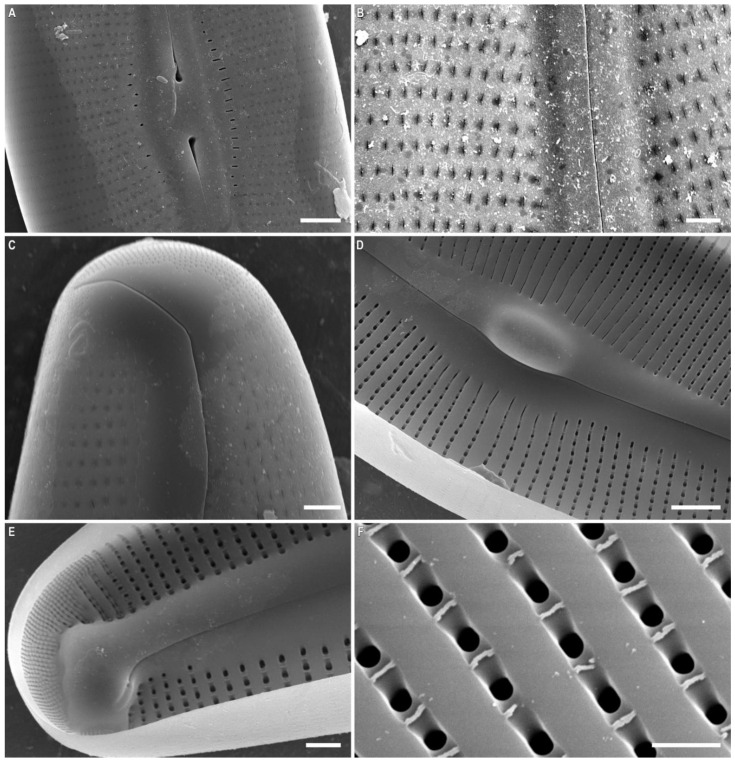
*Cymbella himalaspera* Jüttner and Van de Vijver in Jüttner et al. 2010. Strain B271. Scanning electron microscopy. (**A**–**C**) External views. (**D**–**F**) Internal views. (**A**,**D**) Central area. (**B**,**C**,**E**,**F**) Valve ends. Areolae. Scale bars (**A**,**D**) = 5 μm; (**B**,**C**,**E**) = 2 μm, (**F**) = 1 μm.

*Cymbella himalaspera* Jüttner and Van de Vijver in Jüttner et al. 2010 (Figure 2, Figure 3 and Figure 4).

**Distribution.** Nepal, Everest National Park [9] and Eastern Siberia, Buryatia (strain B271, slide no. B271).

**Description.** LM (Figure 2). Cells solitary. A single chloroplast is present per cell. The chloroplast has two arms, that underlie the valves, and they are connected by a wide isthmus (Figure 2A). Valves dorsiventral, moderately arched with convex dorsal margin and slightly convex ventral margin, at the valve centre. Ends are broadly rounded, not protracted. Length 190.0–201.5 μm, breadth 40.0–42.4 μm. Length-to-breadth ratio 4.6–4.8 (n = 21). Axial area is moderately wide and linear. Central area is shallow, about 1/3 of the valve width. Raphe filiform, lateral [2, p. 324, pl. 67, Figure 3B], proximal raphe ends are weakly deflected ventrally, and tipped with inflated drop-shaped pores. Distal raphe ends are deflected to the dorsal margin. Striae radiate, becoming subparallel at the central part and condensing towards to the ends, are 7–8 in 8 μm at the central part, 9.5–10.5 in 10 μm near the ends. Areolae coarse, 11–12 in 10 μm.

SEM, external view (Figure 3A,B and Figure 4A–C). The slit of the raphe is located on a raised rib. Proximal raphe endings are slightly expanded, drop-shaped, and unilaterally deflected towards the ventral margin. The raphe towards the ends is weakly undulate, unilaterally curved to the side opposite to the proximal raphe ends (dorsally) and extending onto the valve mantle. The raphe fissures are located in small hyaline areas. Apical pore fields consist of very small round porelli. Stigmata are present on the ventral side of the central margin, 13–18, round and slit-like in shape. On the dorsal side of the central area, 9–10 areolae are adjacent to the central area and widened, forming round, elongate or irregular pores.

Striae are uniseriate, extending from the valve face and onto the mantle on both dorsal and ventral margins. Areolar openings are X-, Y-, star- or tree-shaped closer to central part of valve, becoming longitudinal slits towards the apices.

SEM, internal view (Figure 3C and Figure 4D–F). The raphe is arcuate. Proximal raphe endings are located beneath a robust central nodule. Distal raphe ends terminate as large helictoglossae oriented towards the dorsal margin. Areolae are arranged in a narrow series compared to the wide interstriae (virgae) which lack occlusions. Vimines are present. The apical pore field alveoli are radially arranged around the terminal nodule which bears a well-developed helictoglossa.

**Figure 5:**
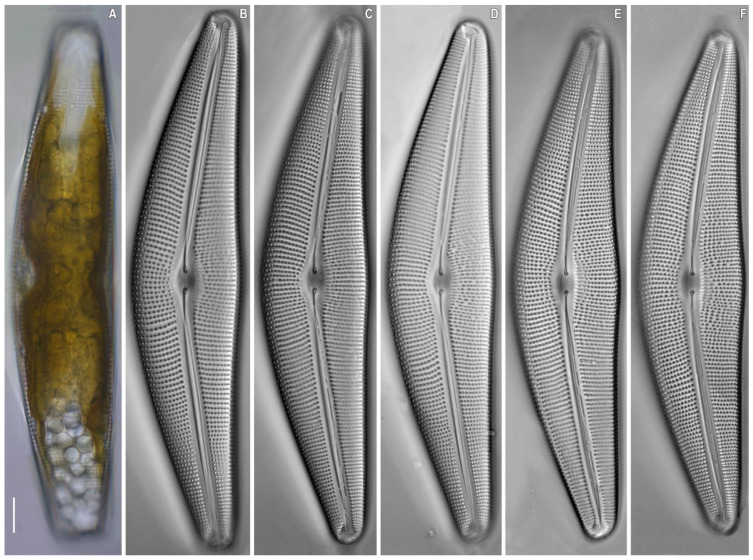
*Cymbella baicalaspera* Glushchenko, Kulikovskiy and Kociolek sp. nov. Strain B207. Light microscopy, differential interference contrast, size diminution series. (**A**) Live cells with chloroplast structure. (**B**–**F**) Oxidized material. Slide No. B207. (**B**) Holotype. Scale bar = 10 μm.

**Figure 6:**
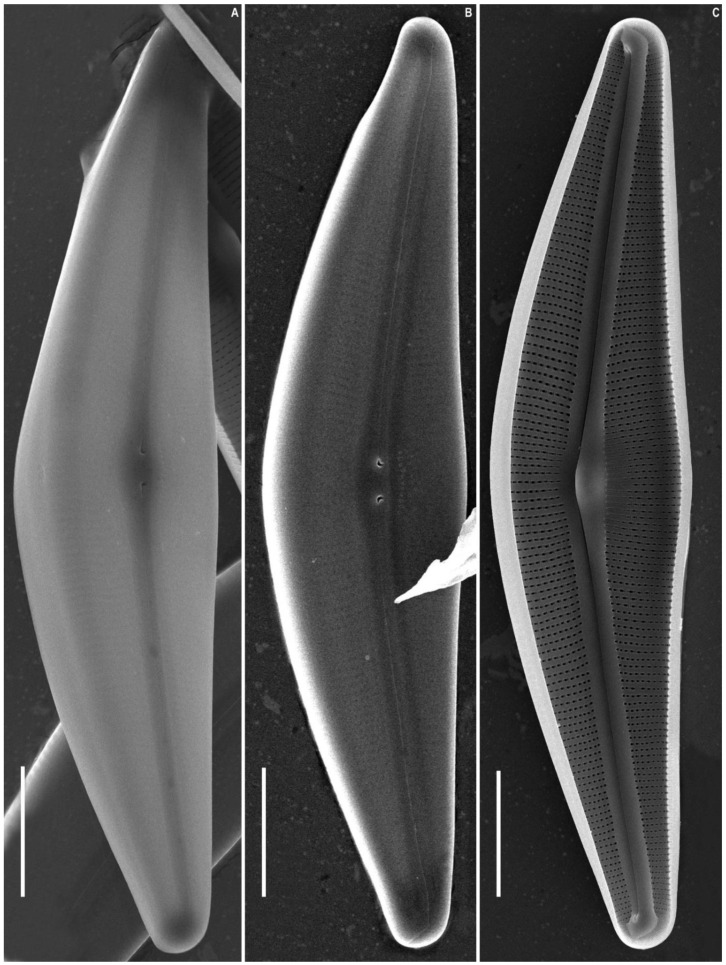
*Cymbella baicalaspera* Glushchenko, Kulikovskiy and Kociolek sp. nov. Strain B207. Scanning electron microscopy. (**A,B**) External views. (**C**) Internal view. Scale bars = 20 μm.

**Figure 7:**
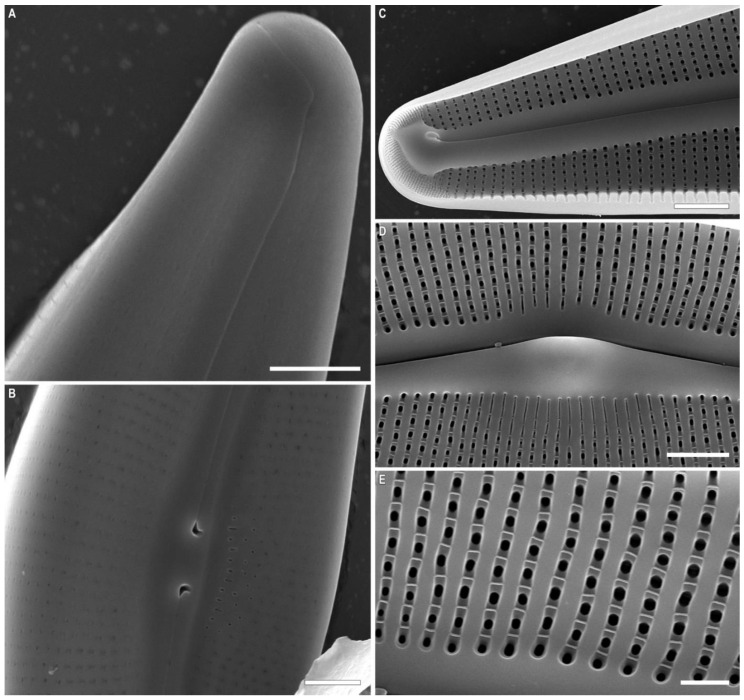
*Cymbella baicalaspera* Glushchenko, Kulikovskiy and Kociolek sp. nov. Strain B207. Scanning electron microscopy. (**A**,**B**) External view. (**C**–**E**) Internal view. (**A**–**D**) Central area. (**E**) Areolae. Scale bars (**A**–**D**) = 10 μm; (**E**) = 2 μm.

**Figure 8:**
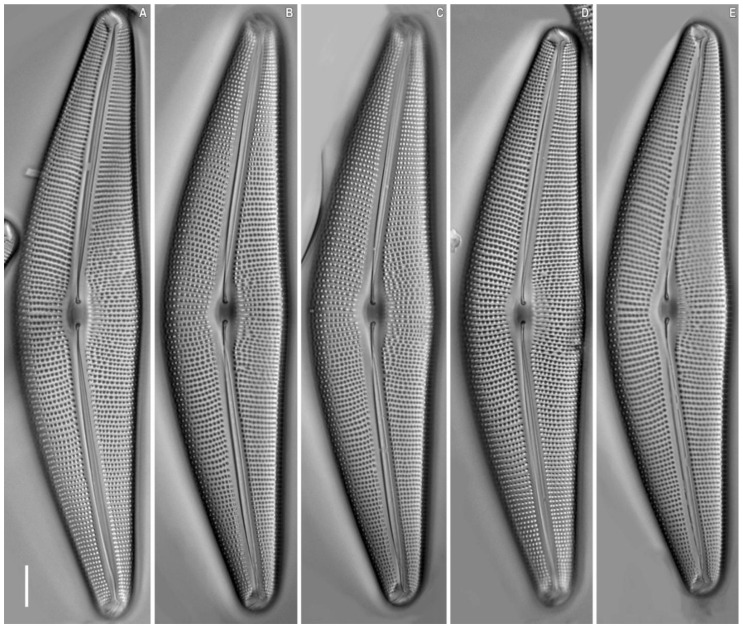
*Cymbella baicalaspera* Glushchenko, Kulikovskiy and Kociolek sp. nov. Strain B290. (**A**–**E**) Light microscopy, differential interference contrast, size diminution series. Slide No. B290. Scale bar = 10 μm.

*Cymbella baicalaspera* Glushchenko, Kulikovskiy and Kociolek sp. nov. (Figure 5, Figure 6, Figure 7 and Figure 8).

**Holotype.** Collection of Maxim Kulikovskiy at the Herbarium of the Institute of Plant Physiology Russian Academy of Science, Moscow, Russia, holotype here designated, slide No. B207 (Figure 5B).

**Type strain.** B207, isolated from the sample 11.2, deposited at the collection of Maxim Kulikovskiy at the Herbarium of the Institute of Plant Physiology Russian Academy of Science, Moscow, Russia.

**Isotype.** Slide no B207a, from oxidized culture strain B207, isolated from sample 195, deposited in the herbarium of MHA, Main Botanical Garden Russian Academy of Science, Moscow, Russia.

**Type locality.** Russia, Zagza River, periphyton, 52°31.656′ N; E107°05.114′ E. Collected by M.S. Kulikovskiy, 15 July 2011.

**Representative DNA sequences for *Cymbella baicalaspera* strains.** Partial 18S rRNA gene sequences comprising V4 domain sequence (GenBank accession numbers: OP070046 for B207, OP070048 for B290) and partial *rbc*L sequences (GenBank accession numbers: OP066547 for B207, OP066548 for B290).

**Representative specimens.** Strain B207 (slide no B207, type strain, Figure 5, Figure 6 and Figure 7); Strain B290 (slide no B290, Figure 8).

**Etymology.** Epithet refers to the Baikal rift zone and similarity to *Cymbella aspera*.

**Distribution.** As yet known only from the type locality.

**Description.** LM (Figure 5 and Figure 8). Solitary cells. A single chloroplast is present per cell. The chloroplast has two arms, which underlie the valves, and they are connected by a wide isthmus (Figure 5A). Valves are dorsiventral, moderately arched with convex dorsal margin and slightly convex ventral margin at the valve centre. Ends are broadly rounded, not protracted. Length 143.4–153.0 μm, breadth 30.6–32.2 μm. Length-to-breadth ratio 4.6–4.8 (n = 24). Axial area is moderately wide and linear. Central area is shallow, about 1/3 of the valve width.

Raphe is filiform, lateral [2, p. 324, pl. 67, Figure 3B], with proximal raphe ends weakly deflected ventrally, terminating as inflated drop-shaped pores. Distal raphe ends are deflected to the dorsal margin. Striae are radiate, becoming subparallel at the central part and condensed towards to the ends, 8–9 in 8 μm at the central part, 11–12 in 10 μm near the ends. Areolae are coarse, 10–11 in 10 μm.

SEM, external view (Figure 6A,B and Figure 7A,B). The raphe slit is located on a raised rib. Proximal raphe endings are slightly expanded, drop-shaped and unilaterally deflected. The raphe towards the ends is weakly undulate, unilaterally curved opposite from the proximal raphe ends, extending onto the valve mantle. The raphe fissures are located in small hyaline areas. Apical pore fields consist of very small round porelli. Stigmata are present on the ventral side of the central margin, there are 8–13, which round to slit-like in shape. On the dorsal side of the central area there are 4–10 areolae adjacent to the central area. The areolae are widened, forming round, elongate or irregular pores. Striae are uniseriate, and extend in from the valve face towards mantle on both dorsal and ventral margins.

Areolae openings are X-, Y-, star- or tree-shaped closer to central part of valve, becoming longitudinal slits towards the valve apices.

SEM, internal view (Figure 6C and Figure 7C–E). The raphe is arcuate. Proximal raphe endings are located beneath a robust central nodule. Distal raphe ends terminate as small helictoglossae. Areolae are arranged in a narrow series as compared to the wide interstriae (virgae). Areolae lack occlusions. Vimines are present. The apical pore field alveoli are radially arranged around the terminal nodule bearing a well-developed helictoglossa.

Another strain *Cymbella baicalaspera* Glushchenko, Kulikovskiy and Kociolek sp. nov. (B290) was isolated from the same sample as the strain B207. Valves of strain B290 (Figure 8) have the following characteristics: length: 93.9–99.7 μm, breadth: 21.0–21.4 μm, density of striae in the central part: −9.0–9.5 in 10 μm, density at the ends: −11.5–12.5 in 10 μm, areolae density: 10–11 in 10 μm.

## 3. Discussion

The valves of *C. himalaspera* from our material are morphologically similar to those from the original material from Nepal (Table 1). However, the outer openings of the stigmoids in valves investigated here are more elongated in our specimens. The inner openings of the stigmoids in our material are noticeably slit-like, while in the material from Nepal they are shorter [11, Figure 3: 6]. We interpret these differences due to intraspecific variation within this taxon.

The newly proposed species, *Cymbella baicalaspera* sp. nov., is morphologically very similar to *C. himalaspera* (Table 1). *Cymbella baicalaspera* sp. nov. differs from the Nepalese specimens of *C. himalaspera* in being more narrow (30.6–32.2 µm in our species versus 35–49 µm in *C. himalaspera*). At the same time, the shape of areola openings in the species is similar (Table 1).

*Cymbella baicalaspera* sp. nov. differs from *C. peraspera* in valve breadth (30.6–32.2 µm in our species versus 44–52 µm in *C. peraspera*), and differs from *C. aspera* in a smaller variety of areola opening types. The density of striae in 10 µm, as well as the areolae, are generally similar (Table 1). *Cymbella baicalaspera* sp. nov. differs from *C. subhimalaspera* in valve width (30.6–32.2 µm in our species versus 21–25 µm in *C. subhimalaspera*).

At the same time, the shape of the areola openings between the two species is similar (Table 1).

**Table 1:** Morphological comparison of *Cymbella baicalaspera* sp. nov., and related species.

**Species/Feature**	*C. baicalaspera* sp. nov.	*C. himalaspera* B271	*C. himalaspera*	*C. aspera*	*C. peraspera*	*C. subhimalaspera*	*C. ameliana*
**Central Raphe Endings**	weakly deflected ventrally, tipped with inflated drop-shaped pores	weakly deflected ventrally, tipped with inflated drop-shaped pores	large drop-like, ventrally deflected	moderately large, round, almost straight or slightly ventrally deflected	moderately large, round, almost straight or slightly ventrally deflected	large drop-like, straight or slightly ventrally deflected	are typically crook-shaped and almost entirely curved backwards
**Length, μm**	143.4–153.0	190.0–201.5	150–280	110–200	(130) 154–320	104–133	160–270
**Breadth, μm**	30.6–32.2	40.0–42.4	35–49	26–35	44–52	21–25	40–46
**Length/Breadth Ratio**	4.6–4.8	4.6–4.8	4.1–5.6	max. 5.7	max. 6	4.9–5.8	5.5–6.0
**Striae in 10 μm**	8–9 (11–12 near the ends)	7–8 (9.5–10.5 near the ends)	7–9	6.5–8.0 (10 near the ends)	5–8 (10 near the ends)	8–11 (10–12 near the ends)	7–8 (11–12 near the ends)
**Areolae, Shape**	X-, Y-, star- or tree-shaped closer to central part of valve, becoming longitudinally-slits to the valve ends	X-, Y-, star- or tree-shaped closer to central part of valve, becoming longitudinally-slits to the valve ends	X-, Y-, star-, tree-shaped	I-shaped	I-shaped	X-, I-shaped, oval, curved	X-, I-shaped, slit-like weakly curved
**Areolae in 10 μm**	10–11	11–12	10–12	8–10 (11)	7–10 (11)	10–12 (15 near the ends)	10–11
**Stigmata**	8–13, round and slit-like	13–18, round and slit-like	large number, round to slightly elongate	7–10	large number, round	8–11, elongate	large number, small round
**Reference**	This study	This study	[11]	[2,11]	[2,11]	[2,11]	[10]

*Cymbella baicalaspera* sp. nov. differs from *C. aspera*, in general, in a higher density of striae of 10 µm: 8–9 in the central part and 11–12 at the ends in our species versus 6.5–8.0 in the central part, up to 10 at the ends in *C. aspera*. The variety of areola shapes in our species is also higher (Table 1). *Cymbella baicalaspera* sp. nov. differs from *C. amelieana*, in general, in the tear-shaped ends of the central branches of the raphe, while in *C. amelieana* the valve ends are hook-shaped. The length/width ratio of our species is less than that of *C. amelieana* (4.6–4.8 versus 5.5–6.0).

According to the molecular phylogeny presented here, our two investigated species belong to the *Cymbella aspera*-group. This group is characterized by very large-sized valves and the presence of many stigmoids. The *Cymbella himalaspera* strain from Baikal is closely related to our new species, *Cymbella baicalaspera* sp. nov., as indicated in the molecular-based phylogeny. These species are also similar morphologically, suggesting they are sister taxa and a case of sympatric speciation in the ancient Lake Baikal area [25].

Specimens of *C. himalaspera* in our material do not differ substantially from the type specimens from Nepal. This suggests that Lake Baikal diatoms are more widespread and not restricted to ancient Lake Baikal, a topic we discussed previously [25]. Further research is necessary to assess proportions of diatom species endemic exclusively to Lake Baikal versus the broader Transbaikal region.

In many cases, invasion of diatoms from surroundings area to Lake Baikal have occurred, resulting in species flocks. We believe that the two giant taxa discussed here are not the only large species present in this system, and that more careful investigation of this complex of interesting species in Lake Baikal and surroundings areas are required.

## 4. Materials and Methods

Sampling. The samples used in the present report were collected from Eastern Siberia, Russia by M.S. Kulikovskiy. Water mineralization and temperature measurements were performed using the Hanna Combo (HI 98129) multiparameter probe (Hanna Instruments, Inc., Woonsocket, RI, USA). A list of all strains examined in this study with their GenBank accession numbers and geographic location of sampling sites with measured ecological parameters is presented in Table 2.

**Culturing.** A subsample of each collection was added to WC liquid medium [26]. Monoclonal strain was established by micropipetting single cell under an inverted microscope. Non-axenic unialgal cultures were maintained in WC liquid medium at 22–25 °C in a growth chamber with a 12:12 h light:dark photoperiod.

**Preparation of slides and microscope investigation.** The cultures were treated with 10% hydrochloric acid to remove carbonates and washed several times with deionized water for 12 h. Afterwards, the sample was boiled in concentrated hydrogen peroxide (≈37%) to remove organic matter. It was washed again with deionized water four times at 12 h intervals. After decanting and filling with deionized water up to 100 mL, the suspension was pipetted onto coverslips and left to dry at room temperature. Permanent diatom preparations were mounted in Naphrax^®^ (Brunel Microscopes, Chippenham, UK).

Light microscopic (LM) observations were performed with a Zeiss Axio Scope A1 microscope equipped with an oil immersion objective (×100, n.a. 1.4, differential interference contrast [DIC]) and Axiocam ERc 5s camera (Zeiss, Oberkochen, Germany). Valve ultrastructure was examined by means of scanning electron microscopes JSM-6510LV (IBIW, Institute for Biology of Inland Waters RAS, Borok, Russia).

For scanning electron microscopy (SEM), part of the suspensions was fixed on aluminum stubs after air-drying. The stubs were sputter-coated with 50 nm of Au by means of a Eiko IB 3 (Eiko Engineering, Yamazaki, Hitachinaka Shi, Ibaraki Ken, Japan). The suspension of cleaned material and slides were deposited in the collection of Maxim Kulikovskiy at the Herbarium of the Institute of Plant Physiology Russian Academy of Sciences, Moscow, Russia.

### Molecular Methods

Total DNA of monoclonal cultures was extracted using Chelex^TM^ 100 Molecular Biology Grade Resin (Bio-Rad, Hercules, CA, USA) according to the manufacturer’s protocol 2.2. Partial 18S rDNA (406–431 bp, including V4 domain), and partial plastid *rbc*L (606–615 bp) genes were amplified using primers D512 for and D978rev from Zimmermann et al. [27] for 18S rDNA fragments and *rbc*L40+ from Ruck and Theriot [28] and *rbc*L1255- from Alverson et al. [29] for *rbc*L fragments.

Amplifications of the 18S rDNA fragments and partial *rbc*L gene fragment were carried out using the premade mix ScreenMix (Evrogen, Moscow, Russia) for the polymerase chain reaction (PCR). The conditions of amplification for 18S rDNA fragments were: an initial denaturation of 5 min at 95 °C, followed by 35 cycles at 94 °C for denaturation (30 s), 52 °C for annealing (30 s) and 72 °C for extension (50 s), and a final extension of 10 min at 72 °C. The conditions of amplification for partial *rbc*L were: an initial denaturation of 5 min at 95 °C, followed by 45 cycles at 94 °C for denaturation (30 s), 59 °C for annealing (30 s) and 72 °C for extension (80 s), and a final extension of 10 min at 72 °C.

The resulting amplicons were visualized by horizontal agarose gel electrophoresis (1.5%), coloured with SYBR Safe (Life Technologies, Carlsbad, CA, USA). Purification of DNA fragments was performed with the ExoSAP-IT kit (Affymetrix, Santa Clara, CA, USA) according to the manufacturer’s protocol. A total of 18S rDNA fragments and partial *rbc*L gene were decoded from two sides using forward and reverse PCR primers and the Big Dye system (Applied Biosystems, Waltham, MA, USA), followed by electrophoresis using a Genetic Analyzer 3500 sequencer (Applied Biosystems).

Editing and assembling of the consensus sequences were carried out by comparing the direct and reverse chromatograms using the Ridom TraceEdit program (ver. 1.1.0) and Mega7 [30]. Newly determined sequences and DNA fragments from 70 other diatoms, which were downloaded from GenBank (taxa and Accession Numbers are given in the tree, Figure 1), were included in the alignments. Five diatom species from Rhopalodiaceae were chosen as the outgroups.

The nucleotide sequences of the 18S rDNA and *rbc*L genes were aligned separately using the Mafft v7 software and the E-INS-i model [31]. For the protein-coding sequences of the *rbc*L gene, we checked that the beginning of the aligned matrix corresponded to the first position of the codon (triplet). The resulting alignments had lengths of 439 (18S rDNA) and 588 (*rbc*L) characters.

The data set was analysed using Bayesian inference (BI) method implemented in Beast ver. 1.10.1 [32] to construct phylogeny. For each of the alignment partitions, the most appropriate substitution model was estimated using the Bayesian information criterion (BIC) as implemented in jModelTest 2.1.10 [33]. This BIC-based model selection procedure selected the following models, shape parameter α and a proportion of invariable sites (pinvar): TIM3+I+G, α = 0.4720 and pinvar = 0.4410 for 18S rDNA gene; HKY+I, pinvar = 0.8460 for the first codon position of the *rbc*L gene; JC for the second codon position of the *rbc*L gene; TVM+I+G, α = 2.1330 and pinvar = 0.2790 for the third codon position of the *rbc*L gene. We used the HKY model of nucleotide substitution instead of TIM3, the F81 model instead of JC, and the GTR model instead of TVM, given that they were the best matching models available for Bayesian inference.

A Yule process tree prior was used as a speciation model. The analysis ran for 15 million generations with chain sampling every 1000 generations. The parameters-estimated convergence, effective sample size (ESS) and burn-in period were checked using the software Tracer ver. 1.7.1 [32]. The initial 25% of the trees were removed, the rest retained to reconstruct a final phylogeny. The phylogenetic tree and posterior probabilities of its branching were obtained on the basis of the remaining trees, having stable estimates of the parameter models of nucleotide substitutions and likelihood. Maximum likelihood (ML) analysis was performed using the program RAxML [34]. The nonparametric bootstrap analysis with 1000 replicas was used. The statistical support values were visualized in FigTree ver. 1.4.4 and Adobe Photoshop CC (19.0).

## Figures and Tables

**Table 2:** List of all strains examined in this study with their GenBank accession numbers.

Strains	Slide No	Sample Locality	Collection of Date	Coordinates	t (°C)	pH	Cond. (μS cm^−1^)	Substratum	GenBank Accession Number, SSU rDNA, Partial	GenBank Accession Number, *rbc*L, Partial
B207	B207	Sample no. 11.2, Eastern Siberia, Buryatia, Zagza River	15 July 2011	52°31.656′ N 107°05.114′ E	14	8.5	40	periphyton	OP070046	OP066547
B271	B271	Sample no. 28.1, Eastern Siberia, Buryatia, unnamed river near the Zarechnyj Village	17 July 2011	52°33.418′ N 107°08.564′ E	15.4	6.1	63	benthos	OP070047	–
B290	B290	Sample no. 11.2, Eastern Siberia, Buryatia, Zagza River	15 July 2011	52°31.656′ N 107°05.114′ E	14	8.5	40	periphyton	OP070048	OP066548

## Data Availability

Not applicable.
